# 602. Pediatric Burn Management with Bromelain Enzymatic Debridement: A Descriptive Single-center Study

**DOI:** 10.1093/jbcr/irag033.406

**Published:** 2026-04-06

**Authors:** Deanna M DeHoff, Courtney Tresslar, Aaron P Lesher, Rohit Mittal, Adria Johnson, Steven A Kahn

**Affiliations:** South Carolina Burn Center at Medical University of South Carolina, Charleston, SC; Medical University of South Carolina, Charleston, SC; Medical University of South Carolina, Charleston, SC; South Carolina Burn Center at Medical University of South Carolina, Charleston, SC; Medical University of South Carolina, Charleston, SC; Medical University of South Carolina, Charleston, SC

## Abstract

**Introduction:**

Approved for commercial use in pediatric patients in August 2024, bromelain-based enzymatic debridement has been studied extensively in adult burn patients but there is less experience in the pediatric population. Recent randomized controlled trial results showed it is safe and effective in children, with shorter time to complete eschar removal and fewer surgical procedures versus standard care. This is a single-center descriptive study of pediatric burn patients treated both in a clinical trial and commercially.

**Methods:**

This was a single-center, retrospective, descriptive study of patients with mixed partial to full-thickness burns under age 18 treated with bromelain-based enzymatic debridement. Patients were treated according to NEXT clinical trial and institutional protocols between September 2021 and July 2025. Demographics, length of stay, readmission, procedural paiN/Anxiety, and wound closure data were collected. Length of stay per %TBSA was reported and benchmarked against Burn Center Quality Program (BCQP) average of 3.3 days per 1% TBSA.

**Results:**

This chart review included 13 patients, aged 2-17 with median age of 11, and 8 (62%) were male. Burn etiologies included flame (6), friction (3), scald (2), and contact (2). Median burn size was 7% TBSA with 1% TBSA full-thickness injury. The enzyme was applied with conscious sedation in the hydrotherapy suite for 8 patients and at bedside for 5. Analgesia included ketamine for 9 (69%) patients and peripheral nerve block for 4 (31%). All 13 received adjunctive narcotics and additional medications for agitation and breakthrough pain during treatment. Post enzymatic debridement, 6 (46%) patients underwent operative closure on the same day, while 7 (54%) underwent closure on post-application day 1. Surgical management included skin cell suspension autograft (11 patients), split thickness skin graft (8 patients), and skin substitutes (4 patients), with some patients receiving multiple grafting techniques. Skin substitutes included allograft and polylactic acid sheets. Average length of stay was 16 days (2.6 days per 1% TBSA; IQR 1, 7.75). One patient was readmitted. No mortalities occurred.

**Conclusions:**

This single-center retrospective review demonstrates that bromelain-based enzymatic debridement is a feasible treatment option for pediatric patients. Patients experienced 21% lower than expected length of stay compared to national standards (2.6 vs 3.3 days per 1% TBSA) and treatment was safe, with no wound-related readmissions or mortality. These preliminary findings support the feasibility of this minimally invasive approach in the pediatric population. Prospective comparative studies are needed to establish patient subsets that benefit most and to establish standardized treatment protocols for broader implementation in pediatric burn care.

**Applicability of Research to Practice:**

Bromelain is a feasible escharectomy option for pediatric burn patients.

**Funding for the study:**

N/A.

